# Ventral cytoskeletal remodeling as a mechanism for oscillatory load-induced Piezo1 overactivation and cancer cell vulnerability

**DOI:** 10.1007/s12551-026-01461-8

**Published:** 2026-09-10

**Authors:** Ivana Pajic-Lijakovic, Milan Milivojevic, Boris Martinac, Peter V. E. McClintock

**Affiliations:** 1 Faculty of Technology and Metallurgy, Department of Chemical Engineering, University of Belgrade, Belgrade, Serbia; 2 Mechanosensory Biophysics Laboratory, Victor Chang Cardiac Research Institute, Sydney, Australia; 3 St Vincent’s Clinical School, Faculty of Medicine, University of New South Wales, Sydney, NSW Australia; 4 School of Physics and Astronomy, Lancaster University, Lancaster, LA1 4YB UK

**Keywords:** Oscillatory mechanical stimuli, Remodeling of ventral cytoskeleton, Activity of Piezo1 channels, Viscoelasticity, Stability of focal adhesion

## Abstract

The limited selectivity of conventional cancer therapies has stimulated interest in alternative approaches that exploit the distinct mechanical properties of cancer cells. Oscillatory mechanical stimuli, including low-intensity ultrasound and cyclic cellular deformation, represent promising non-chemical strategies capable of inducing selective cancer cell death through activation of the mechanosensitive ion channel Piezo1. However, the mechanisms linking external mechanical loading to Piezo1 overactivation remain insufficiently understood. This review presents a unified mechanobiological framework describing how frequency-dependent remodeling of the ventral cytoskeleton regulates Piezo1 activity in mesenchymal-like cancer cells. The heterogeneous and anisotropic organization of the cancer cell cytoskeleton enables specific mechanical responses, including stress localization, domain twisting under oscillatory extension, and domain tilting under ultrasound stimulation. These remodeling processes alter membrane–cytoskeleton coupling, focal adhesion-associated curvature formation, and Piezo1 conformational dynamics. In particular, torsional loading and curvature-dependent modulation of Piezo1 clusters provide potential mechanisms for sustained channel activation and Ca2⁺-mediated apoptosis. The proposed framework demonstrates that the response to oscillatory mechanical stimulation depends on the interplay between loading parameters and the dynamically remodeled mechanical state of the basal membrane–cytoskeleton system. These insights provide a physical basis for developing mechanically selective anticancer strategies by optimizing oscillatory stimuli to exploit cancer cell-specific vulnerabilities while preserving healthy tissues.

## Introduction

Conventional cancer therapies, including chemotherapy and radiotherapy, remain limited by their insufficient selectivity and the emergence of therapy-resistant cancer cell populations. In particular, these treatments can damage healthy tissues while promoting the survival of quiescent (dormant) cancer cells, which exhibit reduced sensitivity to pharmacological interventions and contribute to disease recurrence (Lindell et al. 2023). Furthermore, ionizing radiation may inadvertently enhance cancer cell migration and invasion by inducing remodeling of the tumor microenvironment and epithelial–mesenchymal transition (Moncharmont et al. 2014; Lacombe and Zenhausern 2022). These limitations have stimulated increasing interest in therapeutic strategies that exploit the distinct mechanical responses of healthy cells as opposed to cancer cells. Supporting this concept, cyclic mechanical stretching has been shown to protect healthy fibroblasts from ultraviolet-induced DNA damage under appropriate loading conditions (Nagayama and Fukuei 2020), suggesting that mechanical stimulation may provide a basis for more selective cancer therapies.

Mechanical forces are emerging as powerful regulators of cancer cell fate, particularly through mechanosensitive ion channels such as Piezo1, which are large homotrimeric membrane proteins, with each monomer comprising 2547 amino acids (Coste et al. 2010; Guo and MacKinnon, 2017; Saotome et al. 2018; Dombroski et al. 2021; Yang et al. 2022). The channel oligomer comprises three propeller-like blades extending into the extracellular region, an extracellular cap, an intracellular anchor, three intracellular beam domains, and a central ion-conducting pore (Ridone et al. 2019; Dombroski et al. 2021). In mesenchymal-like cancer cells, Piezo1 overactivation has recently been identified as a key upstream event triggering sustained Ca2⁺ influx and subsequent apoptosis under specific oscillatory mechanical environments. This suggests that mechanical loading regimes may be harnessed as a non-chemical strategy for selectively targeting mechanically vulnerable cancer phenotypes (Azagury et al. 2016; Yang et al. 2020; Yao et al. 2022; Tijore et al. 2024; Federico et al. 2025; Pajic-Lijakovic et al. 2026a). The activity of Piezo1 channels is governed by a coupled interplay between (i) lipid reorganization within the membrane (Ridone et al. 2019; Buyan, et al. 2020; Vaisey and MacKinnon 2026) (ii) the viscoelastic properties of the ventral cytoskeleton (Pajic-Lijakovic et al. 2026a), (iii) the local packing density of Piezo1 channels, and (iv) membrane geometry and surface roughness (Bavi et al. 2016; Pajic-Lijakovic et al. 2026a).

Two distinct classes of oscillatory mechanical stimulation have been shown to induce such effects: (i) low-frequency, low-intensity ultrasound fields, and (ii) oscillatory extension of cell monolayers. Although these modalities operate in different frequency and amplitude regimes and involve fundamentally different modes of force transmission (acoustic–mechanical coupling versus substrate-mediated deformation), they both cause overactivation of Piezo1 channels by remodeling of the ventral cytoskeleton (Yao et al. 2022; Tijore et al. 2021;2024; Pajic-Lijakovic et al. 2026a).

The structural changes of the ventral cytoskeleton under oscillatory mechanical stimuli influence: (i) the distribution of mechanical stress across the ventral stress fiber network (Pajic-Lijakovic et al. 2026a,b), (ii) curvature formation around FAs (Yao et al. 2022; Tijore et al. 2022;2024), (iii) the size and roughness of the curvatures (Yao et al. 2022; Tijore et al. 2024; Pajic-Lijakovic et al. 2026a), and (iv) conformational changes of Piezo1 channels directly through the rearrangement of ventral stress fibers or indirectly by creating a coupling between the footprints of Piezo1 within clusters and the surrounding mesoscopic curved regions around FAs (Bavi et al. 2016; Verkest et al. 2025). Characteristic features of the ventral cytoskeletons of mesenchymal-like cancer cells include their inhomogeneity and anisotropy (Damania et al. 2010; Tijore et al. 2021; Pajic-Lijakovic et al. 2026b). This inhomogeneity and disorder are caused by lower relative proportions of tropomyosin 2.1 and mutations of proteins such as: Rho GTPases, APC, or β-catenin (Damania et al. 2010; Chen et al. 2023; Tijore et al. 2024). Consequently, the ventral cytoskeleton of a cancer cell can be considered as an ensemble of interconnected anisotropic cytoskeletal domains (Pajic-Lijakovic et al. 2026a). The inhomogeneous ventral cytoskeleton leads to an inhomogeneous distribution of mechanical stress across the cytoskeleton structure.

Importantly, the mechanisms of ventral cytoskeletal remodeling differ substantially between the two loading regimes. In ultrasound-mediated stimulation, excitation at high frequencies in the range of 20–40 kHz induces tilting of ventral cytoskeletal domains, resulting in the formation of rough curvatures around FAs (Pajic-Lijakovic et al. 2026a). However, lower-frequency excitation in the range of ${10}^{-2}-{10}^{3}$ Hz does not induce tilting of the ventral cytoskeleton but causes interactions between cytoskeletal domains under the shear stress generated, which can induce domain twisting. The extension of anisotropic viscoelastic systems generates both tensile and shear stresses (Pettermann et al. 2021). Despite these differences, both processes are governed by the intrinsic viscoelasticity of the ventral cytoskeleton (Pajic-Lijakovic et al. 2026a). This viscoelastic response ultimately controls the probability of Piezo1 overactivation and subsequent Ca^2^⁺-induced apoptotic signaling (Tijore et al. 2024; Pajic-Lijakovic et al. 2026a). The main goal of this review is to illuminate the cause-consequence relationship between structural changes of the ventral cytoskeletons in cancer cells and enhanced activity of Piezo1 channels over a wide range of frequencies.

We therefore review a variety of experimental data related to the responses of different cell types to oscillatory mechanical stimuli. Based on this information, we develop a unified physical framework describing ventral cytoskeletal dynamics under diverse oscillatory loading conditions, and we consider their consequences for Piezo1 channel activation. We show that viscoelastic remodeling of the ventral cytoskeleton across a broad frequency spectrum plays a central role in regulating mechanically induced Piezo1 overactivation. Despite extensive studies of cytoskeletal mechanics under static or slowly-varying loads, the response of the ventral cytoskeleton to broadband oscillatory mechanical stimulation remains largely unexplored. This includes regimes relevant to both ultrasound-mediated excitation and oscillatory deformation of epithelial monolayers, where distinct loading mechanisms may nevertheless converge on similar mechanotransductive outcomes.

## Main characteristics of oscillatory mechanical stimuli

The responses of various cell types to: (i) oscillatory in-plane extension in the frequency range of ${10}^{-2}-{10}^{3}$ Hz and (ii) low-frequency and low-intensity ultrasound in the frequency range of 20–40 kHz have been analyzed. Frequencies within the range of ${10}^{-2}-{10}^{3}$ Hz, corresponding to time scales from minutes to milliseconds, have been investigated and utilized with amplitudes of up to 20% strain and exposure times varying from several tens of minutes to several hours (Tijore et al.  HYPERLINK "SPS:refid::bib59" 2021; 2024;; Yao et al. 2022). Oscillatory extension of a cell monolayer is induced by cyclic stretching of the underlying substrate. Cellular structural responses within the frequency range of $1-{10}^{3}$ Hz include: (i) intra and inter-filament interactions (Heinrich and Sackmann 2006), (ii) changes in the bending state of the lipid bilayer (Braunmüller et al., 2012) , and (iii) mechanical coupling between the cytoskeleton and the lipid bilayer (Pajic-Lijakovic and Milivojevic 2015). Mesoscopic structural changes such as cytoskeletal domain rearrangements occur at lower frequencies of approximately $\sim {10}^{-1}$ Hz, whereas actomyosin contractions are associated with even lower frequencies of approximately $\sim {10}^{-2}$ (Gorfinkiel and Blanchard 2011).

At high frequencies in the range of 20–40 kHz and above, intrafilament rearrangements and crosslink dynamics are effectively frozen due to the mismatch between forcing period and molecular relaxation times. In this regime, the ventral cytoskeleton responds predominantly as an elastic structure: it behaves either as a homogeneous rigid elastic grid or, in the case of structural heterogeneity, as an ensemble of interconnected rigid domains coupled through focal adhesions and interdomain links (Pajic-Lijakovic et al. 2026a). Mechanical response is therefore governed by elastic deformation and relative motion of preexisting structural units rather than by internal cytoskeletal remodeling. The wavelength of the ultrasound $\lambda =\frac{c}{f}$ in human soft tissue is high and equal to $\lambda =7.5 \mathrm{c}\mathrm{m}$ for frequency $f=20 \mathrm{k}\mathrm{H}\mathrm{z}$. Short wavelength ensures deeper penetration of ultrasound waves into the body. Acoustic energy dissipation per unit propagation distance increases with ultrasound frequency due to stronger absorption and scattering in heterogeneous tissue, leading to more localized energy deposition and reduced penetration depth. The intensity of ultrasound applied as an anticancer therapy is $\le 100 \frac{\mathrm{m}\mathrm{W}}{{\mathrm{c}\mathrm{m}}^{2}}$. Intensity of ultrasound determines the magnitude of the acoustic pressure amplitude equal to: ${p}_{0}=\sqrt{2\rho cI}$ (where I is the intensity of ultrasound, c is the speed of ultrasound within water-like media $\left. c \approx 1500 \frac{\mathrm{m}}{\mathrm{s}}\right)$, ρ is the density of multicellular systems $\rho \approx 1000 \frac{kg}{{m}^{3}}$). For the intensity of $100 \frac{\mathrm{m}\mathrm{W}}{{\mathrm{c}\mathrm{m}}^{2}}$, the pressure amplitude is 55 kPa. The intensity and frequency of ultrasound influence the resultant displacement amplitude: $\Delta {r}_{LIUS}=\sqrt{\frac{2I}{\rho c{\omega }^{2}}}$. Smaller displacement fields (< 30 nm) typically induce reversible elastic vibrations of the ventral cytoskeleton, as deformation remains within the linear response regime of a connected actin network. In contrast, larger displacement fields (> 100 nm) can generate sufficient strain localization and interdomain shear to trigger irreversible structural rearrangements, particularly in inhomogeneous ventral cytoskeletons of mesenchymal-like cancer cells where mechanical connectivity is spatially heterogeneous. These irreversible structural rearrangements can be related to the overactivation of Piezo1 channels, is in accordance with the findings of Tijore et al. (2024). They pointed out that ultrasound with an intensity of 39 mW·cm⁻^2^ and a frequency of 33 kHz triggers calcium-induced apoptosis of cancer cells, while ultrasound at the higher frequency of 120 kHz does not have this effect. Epithelial cells and fibroblasts retained their viable state. An increase in frequency from 33 to 120 kHz for the same intensity of ultrasound of 39 mW cm⁻^2^ causes a decrease in the displacement amplitude from 110 to 30 nm. To minimize unwanted heating and cavitation, the duty cycle, which is defined as the fraction of time the ultrasound is turned on, should be kept below 50%. Depending on the specific application, exposure times typically range from a few minutes to several hours. Further consideration requires discussion of the responses of different cell types to oscillatory mechanical stimuli.

## Cell responses to oscillatory mechanical stimuli

Oscillatory mechanical stimulation over a broad range of frequencies elicits distinct, cell type–dependent responses. Mesenchymal-like cancer cells are particularly susceptible, undergoing apoptosis under both intermediate-frequency (10⁻^2^–103 Hz) in-plane uniaxial oscillatory extension (Zhang et al. 2006; Spencer et al. 2021; Tijore et al. 2021; Yao et al. 2022) and high-frequency (20–40 kHz) ultrasound stimulation (Tijore et al. 2024). Several studies have shown that, in both cases, the apoptotic response of cancer cells is associated with overactivation of Piezo1 mechanosensitive channels, which promote calcium influx and activate a calpain-dependent mitochondrial pathway leading to apoptosis (Tijore et al. 2021, 2024; Singh et al. 2021; Yao et al. 2022). Cancer cells are more vulnerable to low-intensity ultrasound in the frequency range of 20–40 kHz (Singh et al. 2021; Tijore et al. 2024) than at higher frequencies of MHz (Federico et al. 2025; Azagury et al. 2016; Bai et al. 2012). It is in accordance with the fact that lower frequency-ultrasound generates higher displacement amplitudes that can induce intensive structural changes of the ventral cytoskeleton. The phenomenon will be elaborated in more detail in the section.

In contrast, epithelial cells and fibroblasts remain viable under similar mechanical conditions and can even exhibit enhanced growth and migratory activity (Tijore et al. 2021;2024). These contrasting outcomes suggest fundamentally different mechanotransduction pathways across different cell types, governed by their underlying cytoskeletal organization and mechanical coupling properties between the ventral cytoskeleton, focal adhesions, and cell membrane. A summary of the responses of various cell types under oscillatory extension and ultrasound loading is shown in Table 1:

**Table 1:** Summary of the responses of different types of cells under oscillatory extension and ultrasound loading

Cell type	Frequency	Characteristics of mechanical stimulus	Outcome	Reference
Tumor cell lines (MDA-MB-231, A375p, HT1080) vs normal epithelial (MCF10A, HFF)	33 kHz; 120 kHz	Pulsatile 39 mW·cm⁻^2^ 50% duty cycle 2 h exposure time	At 33 kHz: Piezo1 overactivation-induced cancer cell apoptosis; at 120 kHz: no increase in apoptosis Healthy epithelial cells and fibroblasts were unaffected	Tijore et al. (2024)
PANC-1 cells (i.e., epithelioid carcinoma cell line derived from the human pancreas)	30–40 kHz 1 MHz	Continuous < 100 mW·cm⁻^2^ 10- to 30-min exposure time	Inhibition of collective cell migration with effects seen for 48–72 h after irradiation	González et al. (2023)
Breast cancer mesenchymal-like MDA-MB-231 Breast epithelial MCF10A cells A375p (metastatic melanoma)	33 kHz	Pulsatile 7 mW·cm⁻^2^ 50% duty cycle 2 h exposure time	Piezo1 overactivation induced cancer cell apoptosis; Healthy epithelial cells were unaffected	Singh et al. (2021)
Breast epithelial MCF10A cells Breast cancer epithelial-like MCF7 cells	From 400 to 650 kHz	50, 100, and 500 mW·cm⁻^2^	MCF10A epithelial cells remain proliferative, whereas MCF7 cancer cell viability decreases above 550 kHz. The Young’s modulus of MCF7 cells increases by ~ 70% between 400 and 480 kHz, then declines at higher frequencies	Ivone et al. (2019)
MCF10A MDA-MB-231 SKOV3 HT1080 HEK293T	${10}^{-1}$ Hz	5% amplitude strain 2 h exposure time	Calcium induced apoptosis of cancer cells (MDA-MB-231, SKOV3, HT1080, HEK293T) caused by Piezo1 overactivation Healthy epithelial MCF10A cells were unaffected	Tijore et al. (2021; 2022)
HCT116 HT29	${10}^{-1}$ Hz	10% amplitude strain	highly aggressive HCT116 colorectal cancer cells invasion is enhanced; less invasive HT29 is unaffected	Strelez et al., (2021)
HUVECs MDA-MB-231 MCF-7	$1-{10}^{3}$	7.5% and 15% amplitude strain	A decrease in expression of genes linked to proliferation of MDA-MB-231, other cell types were less affected	Spencer et al. (2021)

Tijore et al. (2024) demonstrated that pulsatile low-intensity ultrasound (33 kHz, 39 mW·cm⁻^2^, 50% duty cycle, 2 h exposure) induces extensive apoptosis in cancer cells, including MDA-MB-231, A375p, and HT1080. Using similar ultrasound parameters, Singh et al. (2021) reported comparable findings, showing that apoptosis is mediated through a calpain-dependent mitochondrial pathway initiated by Ca^2^⁺ influx via mechanosensitive Piezo1 channels. In contrast, this pathway was not activated in non-malignant breast epithelial MCF10A cells or fibroblasts, which remained viable. González et al. (2023) investigated the effect of continuous low-intensity ultrasound (frequencies near 1 MHz, intensities below 100 mW·cm⁻^2^) on the collective migration of PANC-1 pancreatic carcinoma cells during wound healing. Exposure for 10–30 min produced a sustained inhibition of collective migration that persisted for 48–72 h after treatment. Similarly, Ivone et al. (2019) reported a significant reduction in the viability of breast cancer MCF7 cells following exposure to low-intensity ultrasound at frequencies above 550 kHz and intensities of 50–500 mW·cm⁻^2^.

Oscillatory extension of epithelial MCF10A and cancerous MDA-MB-231 monolayers (0.5 Hz, 5% strain, 6 h on a rigid substrate) produces contrasting proliferative responses, including a 2.5-fold reduction in the proliferation rate of MDA-MB-231 cells and an increased proliferation rate in MCF10A epithelial cells (Tijore et al. 2021). A similar trend was observed at 1 Hz under otherwise comparable conditions (Tijore et al. 2021). Spencer et al. (2021) further showed that cyclic stretching (1 Hz, 7.5% amplitude) suppresses the expression of proliferation-associated genes in MDA-MB-231 cells relative to static controls. Comparable mechanosensitive behavior is also reported for SKOV3 (human ovarian adenocarcinoma), HT1080 (human fibrosarcoma), and HEK293T (transformed human embryonic kidney) cells, which respond similarly to MDA-MB-231 cells under identical loading conditions (Tijore et al. 2021). In contrast, epithelial-like colorectal cancer cells, including HCT116 and HT29, exhibited either enhanced invasion or no significant change in invasion under cyclic stretching, unlike the mesenchymal-like MDA-MB-231 cells (Strelez et al. 2021). This divergence in mechanobiological response across cancer cell types is attributable mainly to structural differences in the ventral cytoskeleton between epithelial-like and mesenchymal-like phenotypes, as will be discussed below in the section.

To better understand the selective mechanically induced apoptosis of cancer cells, despite epithelial cells and fibroblasts remaining intact or even exhibiting enhanced growth and migration, it is necessary to identify the physical mechanisms linking perturbation to Piezo1 activation in various frequency regimes. Within our framework, mechanical load redistributes mechanical stresses through the ventral cytoskeleton and FA network, thereby modulating local viscoelastic resistance and membrane curvature in FA–associated regions. Because Piezo1 activity is sensitive to membrane mechanics and curvature formation-dependent mechanical cues, these induced structural changes may differentially regulate Piezo1 recruitment and activation in epithelial and mesenchymal-like cancer cells. The following section discusses the principal physical factors governing Piezo1 behavior under low-frequency and high-frequency mechanical load.

## Piezo1 activity within the basal membrane–cytoskeleton system

Piezo1 activity reflects the coupling between channel conformation and the mechanical state of the basal membrane–cytoskeleton system. Piezo1 retains a gating-competent conformation as long as the channel maintains its preferred outward membrane footprint (Bavi et al. 2016). However, the coupling between channel conformation and the mechanical state of the basal membrane–ventral cytoskeleton system can trigger various mechanical scenarios that perturb the stability of this footprint and thereby either enhance or reduce channel activity (Pajic-Lijakovic et al. 2026a,b).

In the absence of pronounced membrane curvatures around FAs, external oscillatory mechanical stimuli can induce cellular mechanical responses, including cytoskeletal rearrangements and changes in Piezo1 activity (Tijore et al. 2021, 2022; Yao et al. 2022). Based on these findings, we propose that such mechanical perturbations may alter stress transmission through the ventral cytoskeleton and modify the coupling of the lipid bilayer to the cytoskeleton, thereby influencing the conformational state and activity of Piezo1 without necessarily promoting channel clustering.

External mechanical stimuli may also affect the formation of mesoscopic membrane curvatures around FAs through the mechanical coupling between FAs, the ventral cytoskeleton, and the substrate matrix (Verkest et al. 2025; Pajic-Lijakovic et al. 2026a). FAs transmit forces generated by the cytoskeleton and external loading to the membrane, whereas the viscoelastic response and degree of homogeneity of the ventral cytoskeleton determine the resistance to membrane deformation (Pajic-Lijakovic et al. 2026b). Consequently, membrane curvatures around FAs are not universal features but emerge only under specific mechanical conditions (Yao et al. 2022; Tijore et al. 2024).

When curvature develops, membrane geometry becomes an additional regulator of Piezo1 activity (Ridone et al. 2019; Verkest et al. 2025; Vaisey and MacKinnon 2026). Curved membrane regions can promote the redistribution and clustering of Piezo1 channels, giving rise to mechanical coupling between neighboring channel footprints (Grage et al. 2011; Verkest et al. 2025). Such footprint coupling may induce transitions between preferred outward and inward footprint states, thereby modifying channel conformation and channel activity depending on: (i) the packing density of channels in a cluster and (ii) the roughness of mesoscopic curvatures (Bavi et al. 2016; Pajic-Lijakovic et al. 2026a). In both the curvature-independent and curvature-dependent regimes, local lipid composition, including cholesterol and anionic lipids, further modulates Piezo1 activity by stabilizing specific footprint geometries and conformational states (Ridone et al. 2019; Vaisey and MacKinnon 2026).

Therefore, understanding the impact of external mechanical stimuli on Piezo1 activity requires consideration of the structural and mechanical properties of the ventral cytoskeleton (Pajic-Lijakovic et al. 2026a). In particular, the organization of the ventral cytoskeleton varies between different cell types, leading to distinct patterns of stress transmission and force redistribution within the basal membrane–cytoskeleton system. Moreover, the viscoelastic properties of the ventral cytoskeleton determine how mechanical stresses are propagated, relaxed, and resisted, thereby influencing both cytoskeletal rearrangements and the potential formation of membrane curvature. Consequently, the main characteristics of the ventral cytoskeleton in various cell types, together with its viscoelastic behavior, must be considered to understand how external mechanical stimuli regulate Piezo1 activity.

## Characteristics of the ventral cytoskeleton of various cell types

Ventral stress fibers are composed of bundles of actin filaments and non-muscle myosin II filaments, which are interconnected and cross-linked by α-actinin and various other actin-binding proteins (Tojkander et al. 2012; Hohmann and Dehghani, 2019; Rajan et al. 2023). This structural interconnection facilitates the sliding of myosin along the actin filaments. Mature ventral stress fibers are made up of 10 to 30 actin filaments (Vallenius 2013). These fibers are anchored at both ends to FAs, thereby linking adjacent FAs across the ventral surface of the cell (Vallenius 2013). As a result, in the ventral cytoskeletal network, FAs serve as mechanical junctions that integrate the contractile stress fiber bundles into a cohesive, load-bearing system (Hohmann and Dehghani, 2019). The number of mature FAs varies according to cell type and matrix stiffness, typically lying in the range of several tens of them (Qin et al. 2015). The behavior of stress fibers is akin to that of rigid chains.

**Fig. 1:**
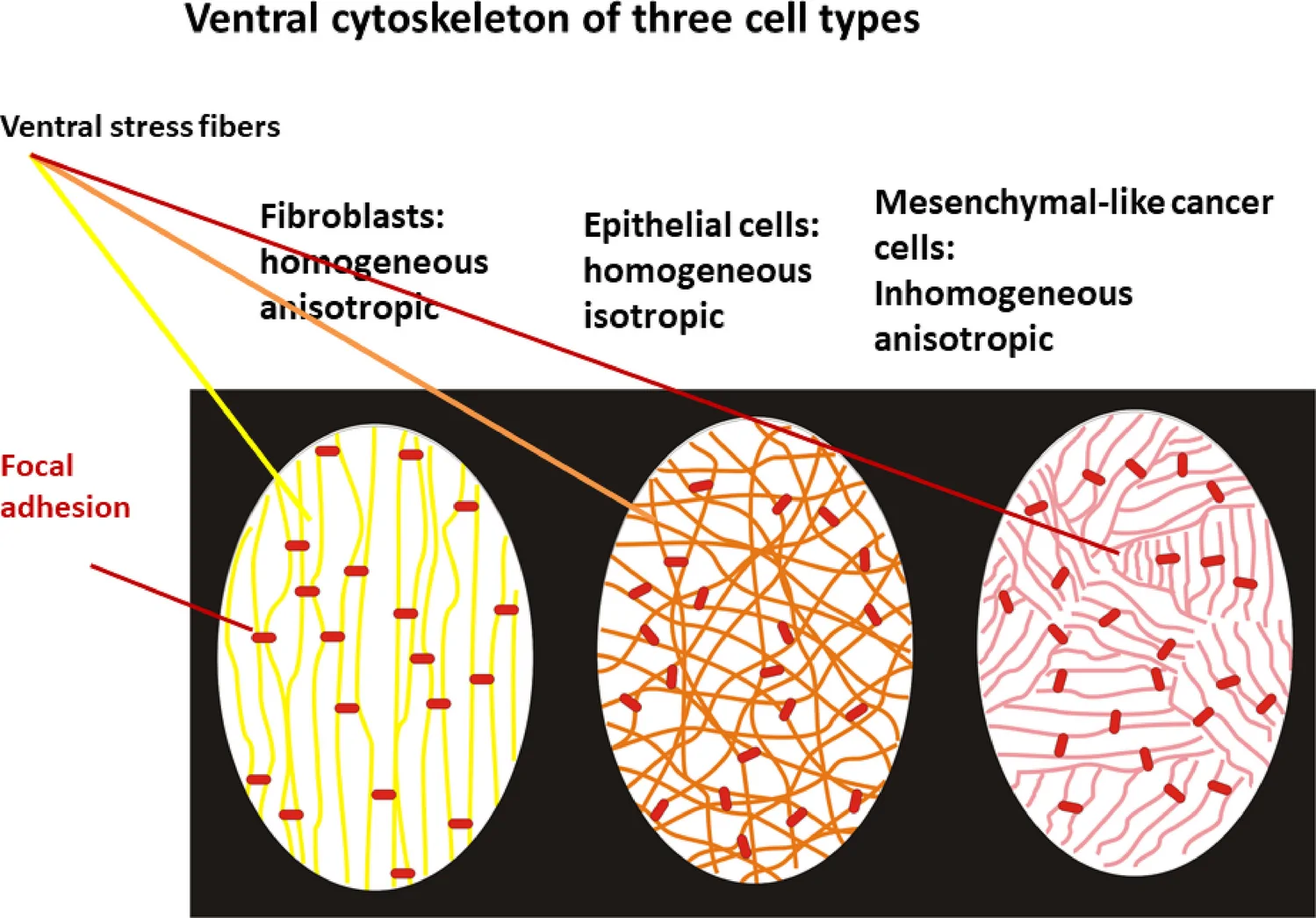
A schematic representation of the ventral cytoskeleton for various cell types. For epithelial cells it exhibits a homogeneous and isotropic structure, whereas in fibroblasts, it is characterized as homogeneous yet anisotropic. In contrast, the ventral cytoskeleton of mesenchymal-like cancer cells displays an inhomogeneous and anisotropic structure

The ventral cytoskeleton exhibits distinct organizational states across different cell types, which determine how mechanical stress is transmitted across the basal membrane–cytoskeleton system. A schematic representation of the ventral cytoskeleton for various cell types is shown in Fig. 1:

In epithelial cells, the ventral cytoskeleton is typically homogeneous and isotropic, leading to broadly distributed and smoothly varying stress fields (Hotulainen and Lappalainen 2006; Jalal et al. 2019). In fibroblasts, the cytoskeletal organization is homogeneous and anisotropic due to the presence of aligned, long stress fibers and directional force transmission (Jalal et al. 2019). When the ventral cytoskeleton is homogeneous, it can be described as a single mechanically coherent domain without internal spatial partitioning. In this case, the stress fiber network behaves as a unified structure. The ventral cytoskeleton for mesenchymal-like cancer cells is highly heterogeneous and anisotropic (Damiania et al., 2010; Pajic-Lijakovic et al. 2026a) and can be regarded as an ensemble of weakly connected mesoscopic domains (Fig. 1). These domains exhibit distinct mechanical properties, orientations, and relaxation times, resulting in spatially non-uniform transmission and redistribution of mechanical stresses across the basal cell surface. Interaction between these domains can influence the activity of Piezo1 channels depending on the frequency range and type of external mechanical stimuli (Pajic-Lijakovic et al. 2026a). In an isotropically organized ventral cytoskeleton, as in the case of epithelial cells, stress fibers lack a preferred orientation, and therefore, no meaningful concept of alignment with the external mechanical load can be defined (Jalal et al. 2019). In contrast, in a homogeneous anisotropic state, long stress fibers are already pre-aligned with the direction of the applied mechanical load, and the response is dominated by their collective reorientation and stretching rather than by reorganization. When the ventral cytoskeleton is domain-structured and anisotropic, each domain can undergo its own internal deformation and cooperative reorientation, and coupling between domains is the main factor responsible for the response of the ventral cytoskeleton under various external mechanical stimuli (Pajic-Lijakovic et al. 2026a).

To understand the frequency-dependent response of the ventral cytoskeleton, particularly in a range of different frequency regimes, it is necessary to consider its viscoelastic properties in greater detail. These responses depend strongly on the frequency-dependent mechanism of energy storage and dissipation under external mechanical stimuli.

### Viscoelasticity of ventral cytoskeleton

An isolated stress fiber is relatively stiff, with a Young modulus of 1–2 MPa (Deguchi et al. 2006) and it behaves as a viscoelastic solid. Its viscoelasticity satisfies the conditions: (i) stress can relax under constant strain and (ii) strain can relax under constant stress. The stress relaxation time is a few seconds (Matsui et al. 2009), while the strain relaxation time (i.e., retardation time) is 4–10 s (Kumar et al. 2006). Kumar et al. (2006) pointed out that strain relaxes exponentially and proposed the linear Kelvin-Voigt model to describe strain relaxation under constant stress conditions. The viscoelasticity of a ventral stress fiber network is a hierarchical combination of both the intrinsic viscoelastic properties of single fibers and the structural coupling/interconnections among those fibers. Ventral stress fibers do not run perfectly parallel without touching; they are heavily coupled side-to-side by interstitial crosslinks and by branching junctions. When one fiber is stretched, it exerts a shear force on its neighbors. The cumulative effects of viscous friction generated by fibers sliding past each other results in energy dissipation during rearrangement of the ventral stress fiber network. Its Young modulus is significantly lower than that of individual stress fibers and is equal to 10–50 kPa (Paul et al. 2008). The relaxation and retardation times increase from several seconds to several tens of seconds during network remodeling (Stachowiak and O’Shaughnessy 2008).

Following these findings from the literature, the Zener constitutive model can be proposed for describing the viscoelasticity of the ventral stress fiber network. This linear model, suitable for viscoelastic solids, satisfies the conditions that (i) stress can relax under constant strain conditions and (ii) strain can relax under constant stress conditions. Stress fiber alignment increases the degree of anisotropy, resulting in higher mechanical stress generation than in isotropic cytoskeleton. The Zener model for anisotropic systems can be expressed as (Pajic-Lijakovic et al 2026c):

1 $${\widetilde{\sigma }}_{V}+{\tau}_{1}{\dot{\widetilde{\sigma }}}_{V}={{\widetilde{\sigma }}^{0}}_{V}\left({\widetilde{\varepsilon }}_{V},{\dot{\widetilde{\varepsilon }}}_{V}\right)+\Delta {\widetilde{\sigma }}_{V}\left({\widetilde{\varepsilon }}_{V},{\dot{\widetilde{\varepsilon }}}_{V}\right)$$

where ${\widetilde{\sigma }}_{V}\left(r,t\right)$ is the stress of the ventral cytoskeleton, ${\dot{\widetilde{\sigma }}}_{V}$ is the rate of stress change equal to ${\dot{\widetilde{\sigma }}}_{V}=\frac{d}{dt}{\widetilde{\sigma }}_{V}$, ${{\widetilde{\sigma }}^{0}}_{V}$ is the isotropic stress equal to ${{\widetilde{\sigma }}^{0}}_{i}\left({\widetilde{\varepsilon }}_{V},{\dot{\widetilde{\varepsilon }}}_{V}\right)=E{\widetilde{\varepsilon }}_{V}+E{\tau}_{2}{\dot{\widetilde{\varepsilon }}}_{V}$**,** E is the modulus of elasticity for the isotropic system, ${\widetilde{\varepsilon }}_{V}\left(r,t\right)$ is the strain equal to ${\widetilde{\varepsilon }}_{V}=\frac{1}{2}\left(\overrightarrow{\nabla }\overrightarrow{u}+{\overrightarrow{\nabla }\overrightarrow{u}}^{T}\right)$**,** $\overrightarrow{u}$ is the local displacement field of the ventral cytoskeleton, $\dot{{\widetilde\varepsilon}_V}={\textstyle\frac{d{\widetilde\varepsilon}_V}{dt}}$, ${\tau}_{1}$ and ${\tau}_{2}$ are the stress relaxation time and strain relaxation time, respectively, $\Delta {\widetilde{\sigma }}_{V}$ is the anisotropic contribution to stress equal to $\Delta {\widetilde{\sigma }}_{V}\left({\widetilde{\varepsilon }}_{V},{\dot{\widetilde{\varepsilon }}}_{V}\right)={\widetilde{\sigma }}_{V}-\frac{1}{2}tr\left({\widetilde{\sigma }}_{V}\right)\widetilde{I}$*,* $\widetilde{I}$ is the unit tensor, $\alpha \left(r,t\right)$ is the degree of anisotropy equal to $\alpha \left(r,t\right) =\frac{1}{N}\sum_{k=1}^{N}\mathrm{c}\mathrm{o}\mathrm{s}{\theta}_{k}^{in-plane}$, and $N\left(r,t\right)$ is the number of stress fibers in the *i*th domain of the ventral cytoskeleton. The degree of anisotropy lies in the range $\alpha \in \left[\mathrm{0,1}\right]$. When α=0, the system is isotropic, while for α=1, the stress fibers are fully aligned.

Model Eq. (1) can be transformed from the time domain into the frequency domain using the Fourier integral transform. The transformed equation can be expressed in the form $F\left[{\widetilde{\sigma }}_{V}\right]={G}_{V}^{*}\left(r,\omega \right)F\left[{\widetilde{\varepsilon }}_{V}\right]$ (where $F\left[\bullet \right]$ is the Fourier transform, ω is the angular velocity, and ${G}_{V}^{*}\left(\omega \right)$ is the complex modulus of the ventral cytoskeleton equal to ${G}_{V}^{*}={G}_{Viso}^{*}+\alpha \Delta {G}_{Vaniso}^{*}$. Here, ${G}_{Viso}^{*}$ is the isotropic contribution equal to ${G}_{Viso}^{*}=E\frac{\left(1+i\omega {\tau}_{2}\right)}{1+i\omega {\tau}_{1}}$, while the anisotropic contribution $\Delta {G}_{Vaniso}^{*}$ is equal to $\Delta {G}_{Vaniso}^{*}=\frac{1}{1+i\omega {\tau}_{1}}\frac{\Delta {\widetilde{\sigma }}_{V}\left(r,\omega \right)}{{\widetilde{\varepsilon }}_{V}\left(r,\omega \right)}$. The complex modulus ${G}_{V}^{*}$ includes storage and dissipated contributions and is equal to ${G}_{V}^{*}={G}_{V}{\prime}+i{G}_{V}^{{\prime}{\prime}}$ (where ${G}_{V}{\prime}\left(r,\omega \right)$ is the storage modulus, ${G}_{V}^{{\prime}{\prime}}\left(r,\omega \right)$ is the loss modulus, and $i=\sqrt{-1}$ is the imaginary unit).

**Fig. 2:**
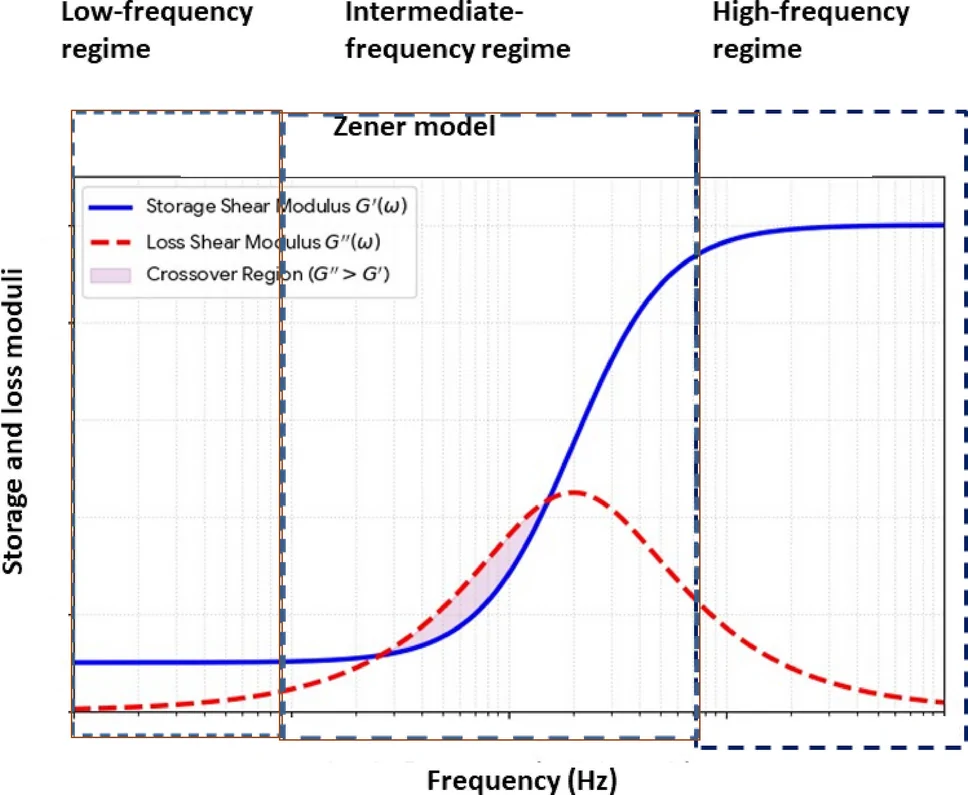
A schematic presentation of the storage and loss moduli as functions of angular frequency for the ventral cytoskeleton, within the Zener model and exhibiting a crossover between the two moduli. Three frequency regimes can be distinguished: (i) low-frequency regime, (ii) intermediate-frequency regime, and (iii) high-frequency regime. While the intermediate-frequency range corresponds to in-plane uniaxial oscillatory extension of the ventral cytoskeleton, the high-frequency range corresponds to ultrasound-induced loading

The storage modulus is equal to ${G}_{V}{\prime}\left(r,\omega \right)=E\frac{1+{\omega }^{2}{\tau}_{1}{\tau}_{2}}{1+{\omega }^{2}{\tau}_{1}^{2}}+\alpha \Delta {G}_{Vaniso}{\prime}$ and the loss modulus is equal to ${G}_{V}^{{\prime}{\prime}}\left(r,\omega \right)=E\frac{\omega \left({\tau}_{2}-{\tau}_{1}\right)}{1+{\omega }^{2}{\tau}_{1}^{2}}+\alpha \Delta {G}_{Vaniso}^{{\prime}{\prime}}$. A schematic representation of the storage and loss moduli vs. angular velocity of ventral cytoskeleton, which corresponds to the Zener model, is shown in Fig. 2:

The dependencies of ${G}_{V}{\prime}\left(r,\omega \right)$, ${G}_{V}^{{\prime}{\prime}}\left(r,\omega \right)$ on ω can be considered within three frequency-regimes:

- **Low-frequency regime** $(\sim {10}^{-3}-{10}^{-1} \mathrm{H}\mathrm{z})$: Storage and loss moduli satisfy the conditions: (i) ${G}_{V}{\prime}\left(r,\omega \right)\sim E+\alpha \Delta {G}_{Vaniso 0}{\prime}$ and ${G}_{V}^{{\prime}{\prime}}\left(r,\omega \right)\sim \omega$ and (ii) ${G}_{V}{\prime}\left(r,\omega \right)>{G}_{V}^{{\prime}{\prime}}\left(r,\omega \right)$. In this regime, the ventral cytoskeleton behaves as viscoelastic solid.
- **Intermediate-frequency regime** $(\sim {10}^{-1}-{10}^{2} \mathrm{H}\mathrm{z})$: Storage and loss moduli satisfy the conditions: (i) ${G}_{V}{\prime}\left(r,\omega \right)\sim {\omega }^{2}$ and (ii) ${G}_{V}^{{\prime}{\prime}}\left(r,\omega \right)$ reach maxima for ${\omega}_{max}=\frac{1}{{\tau}_{1}}$ (where ${\omega}_{max}=2\pi {f}_{max}$). For a ${\tau}_{1}$ of a few seconds, the frequency ${f}_{max}$ corresponds to a few Hz. The energy dissipation during structural rearrangement of the ventral cytoskeleton is pronounced in this regime.

The maximum value of loss modulus ${G}_{V}^{{\prime}{\prime}}\left(r,{\omega}_{max}\right)$ can be either: (i) ${G}_{V}^{{\prime}{\prime}}\left(r,{\omega}_{max}\right)<{G}_{V}{\prime}\left(r,{\omega}_{max}\right)$ for $\frac{{\tau}_{2}}{{\tau}_{1}}<5.83$ indicating solid-like behavior over a wide range of frequencies or (ii) ${G}_{V}^{{\prime}{\prime}}\left(r,{\omega}_{max}\right)>{G}_{V}{\prime}\left(r,{\omega}_{max}\right)$ for $\frac{{\tau}_{2}}{{\tau}_{1}}\ge 5.83$, indicating the solid to liquid state transition. In the second case, when ${G}_{V}^{{\prime}{\prime}}\left(r,{\omega}_{max}\right)>{G}_{V}{\prime}\left(r,{\omega}_{max}\right)$, two crossover angular velocities ${\omega}_{1}=2\pi {f}_{1}$ and ${\omega}_{2}=2\pi {f}_{2}$ should be identified (where ${f}_{1}<{f}_{2}$ and ${f}_{1}<{f}_{max}$, while ${f}_{2}>{f}_{max}$). For these frequencies, storage and loss moduli are equal to each other, i.e., ${G}_{V}{\prime}\left(r,{\omega}_{1}\right)={G}_{V}^{{\prime}{\prime}}\left(r,{\omega}_{1}\right)$ and ${G}_{V}{\prime}\left(r,{\omega}_{2}\right)={G}_{V}^{{\prime}{\prime}}\left(r,{\omega}_{2}\right)$ as is shown schematically in Fig. 2.

- **High frequency regime** $\left(\sim{10}^{2}-{10}^{5}\,\mathrm{Hz}\right)$: Storage and loss moduli satisfy the conditions: (i) ${G}_{V}^{\prime}\left(r,\omega \right)\sim E\frac{\tau_{2}}{\tau_{1}}+\alpha \Delta {G}^{\prime}_{Vaniso\,\infty}$ and ${G}_{V}^{{\prime}{\prime}}\left(r,\omega \right)\rightarrow 0$ and (ii) ${G}_{V}^{\prime}\left(r,\omega \right)>\!\!>{G}_{V}^{{\prime}{\prime}}\left(r,\omega \right)$. In this regime, the ventral cytoskeleton is much stiffer than in the low-frequency regime and it behaves as an elastic rigid grid.

In further work, we will analyze the strain of the ventral cytoskeleton induced by (i) its uniaxial oscillatory extension in the intermediate-frequency range and (ii) its triaxial cyclic compression and extension in the high-frequency regime induced by LIUS.

### Structural rearrangement of ventral cytoskeleton under various mechanical stimuli

**Fig. 3:**
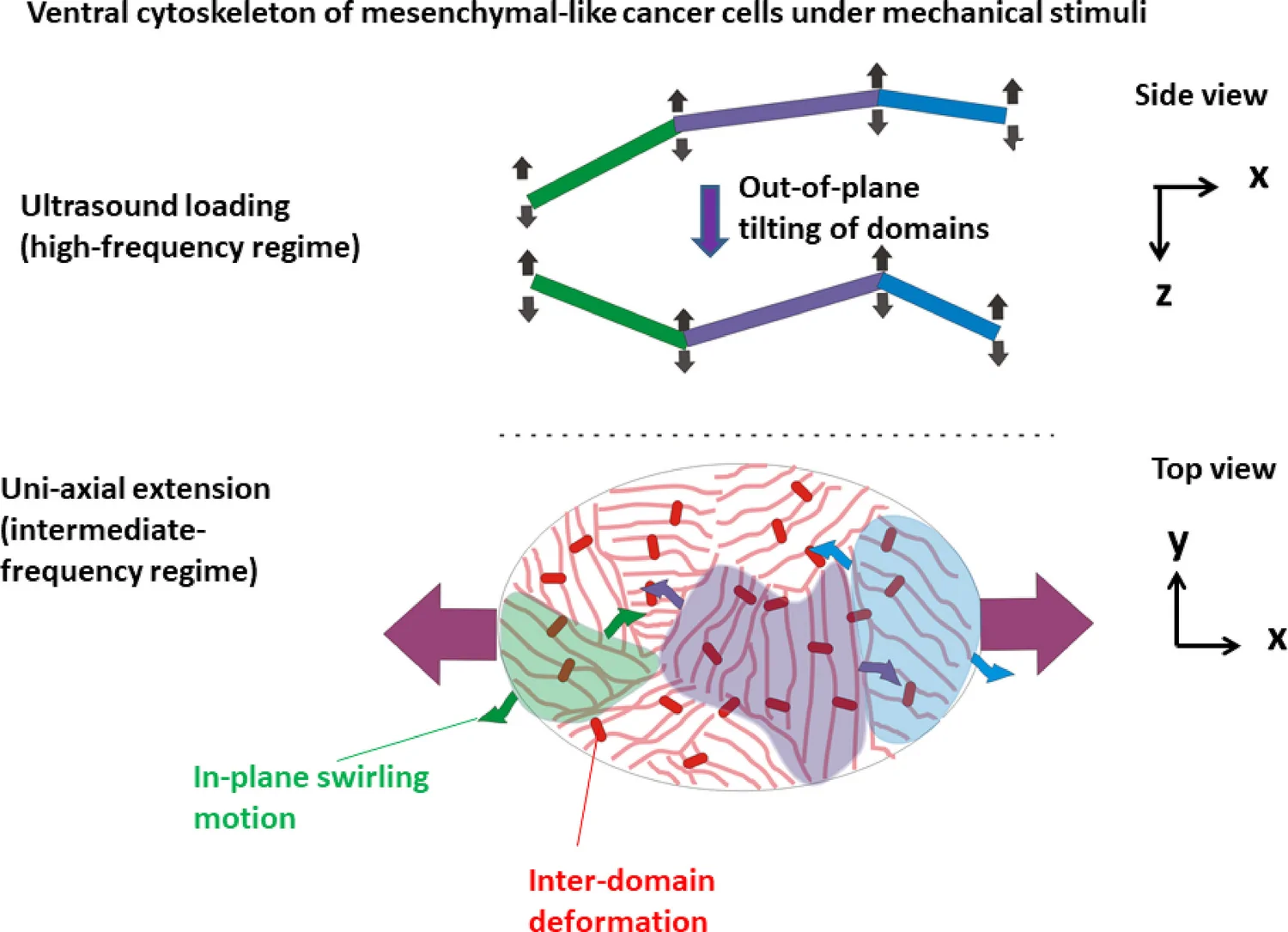
Structural changes in the ventral cytoskeleton of mesenchymal-like cancer cells under: (i) in-plane oscillatory uniaxial extension in the intermediate-frequency regime (bottom) and (ii) ultrasound loading in the high-frequency regime (top) (Yao et al. 2022; Tijore et al. 2024; Pajic-Lijakovic et al. 2026a)

The structural rearrangement of the ventral cytoskeleton depends strongly on frequency. In the intermediate-frequency regime, the ventral cytoskeleton is deformable, whereas in the high-frequency regime it behaves as a rigid grid (Pajic-Lijakovic et al. 2026a). In mesenchymal-like cancer cells, where the ventral cytoskeleton is structurally inhomogeneous and disordered (Damiania et al., 2010), its rearrangement is governed primarily by interactions between cytoskeletal domains. These domains are deformable in the intermediate-frequency regime but become effectively rigid in the high-frequency regime, quantified by the relationship between storage and loss moduli such that ${G}_{V}{\prime}\left(r,\omega \right)\gg {G}_{V}^{{\prime}{\prime}}\left(r,\omega \right)$ (Fig. 2). Emphasis is placed on understanding how inter-domain interactions, under different mechanical stimuli, govern the structural rearrangement of the ventral cytoskeleton in cancer cells. A schematic representation of cytoskeletal structural changes under various oscillatory external stimuli is shown in Fig. 3:

For an inhomogeneous and anisotropic ventral cytoskeleton, oscillatory uniaxial extension can induce (i) deformation between cytoskeletal domains and (ii) in-plane realignment of these domains, resulting in mesoscopic twisting motion. In contrast, the homogeneous, highly aligned stress fiber network of fibroblasts primarily undergoes fiber extension, with little scope for further fiber realignment or mesoscopic twisting. Likewise, the homogeneous and isotropic ventral cytoskeleton of epithelial cells undergoes deformation accompanied by partial alignment of stress fibers in the direction of extension, but does not exhibit mesoscopic in-plane twisting motion.

However, ultrasonic loading in the high-frequency regime can induce a fundamentally different structural rearrangement of the ventral cytoskeleton. In epithelial cells and fibroblasts, ultrasound has little or no effect on the ventral cytoskeleton because the structural rearrangement of stress fibers is effectively frozen at these frequencies. In contrast, in mesenchymal-like cancer cells, inter-domain interactions promote out-of-plane tilting of cytoskeletal domains (Fig. 3). A detailed analysis of the structural rearrangement of the ventral cytoskeleton in different cell types is presented based on the generated strain and stress components, together with the corresponding changes in the degree of anisotropy.

#### Strain components of the ventral cytoskeleton and anisotropy under external in-plane uniaxial extension in intermediate-frequency regime

Uniaxial oscillatory extension of cells in the *x*-direction generates the strain component ${\varepsilon}_{xx}={\varepsilon}_{xx0}\mathrm{s}\mathrm{i}\mathrm{n}\left(\omega t\right)$ (where ${\varepsilon}_{xx0}$ is the amplitude and ω is the angular velocity of oscillations). Normal strain components ${\varepsilon}_{yy}$ and ${\varepsilon}_{xx}$ are related based on the viscoelastic Poisson’s ratio *ν* as: ${\varepsilon}_{yy}=-\upnu {\varepsilon}_{xx}$. Mokbel et al. (2020) conducted measurements of the Poisson's ratio of the actin cortex resulting from the uniaxial compression of an individual HeLa cell. The experimentally determined Poisson's ratio was approximately 0.66, which corresponds to the frequency associated with single-cell contractions (Mokbel et al. 2020). However, Poisson’s ratio ν for a ventral stress fiber network remains experimentally undetermined. When the Poisson’s ratio is ν>0, external uniaxial extension in the *x*-direction induces contraction in the *y*-direction. This extension and compression lead to an increase in the volume of the system when the Poisson’s ratio is ν>0.5 (Pajic-Lijakovic et al. 2025).

Uniaxial extension in the *x*-direction can induce a shear strain component, ${\varepsilon}_{xy}$, particularly in an inhomogeneous and anisotropic ventral stress fiber network of mesenchymal-like cancer cells. However, in fibroblasts containing homogeneous, well-aligned long stress fibers, the shear strain is ${\varepsilon}_{xy}\approx 0$. The shear strain is also ${\varepsilon}_{xy}\approx 0$ in the homogeneous and isotropic ventral cytoskeleton of epithelial cells.

Ventral cytoskeletal domains in cancer cells undergo intra-domain viscoelastic deformation that, within our framework, can be described by a Zener model. Under cyclic uniaxial loading, we propose that shear stress components generate torque-like imbalances within the anisotropic, interconnected network, leading to emergent in-plane twisting of domain orientations (Fig. 3). We further propose that oscillatory extension acts as an energy input to the ventral cytoskeleton, which does not transmit this energy through a purely linear deformation mode. Instead, the heterogeneous and anisotropic network can redistribute the imposed mechanical energy into localized rotational forces, thereby generating in-plane twisting of cytoskeletal domains. We interpret this twisting motion as a physical reorganization mechanism that can increase the degree of anisotropy by progressively aligning stress fiber orientations with the principal deformation direction, thereby reducing the orientational free energy of the cytoskeletal network. The change in the degree of anisotropy proposed here can be expressed as:

2 $$\frac{D\alpha \left(r,t\right)}{Dt}=A{\sigma}_{xy}\left(1-\alpha \right)-\frac{1}{{\tau}_{\alpha }}\alpha +{D}_{\alpha }{\nabla }^{2}\alpha$$

where $\frac{D\alpha }{Dt}$ is the substantial derivative equal to $\frac{D\alpha }{Dt}=\frac{\partial \alpha }{\partial t}+\overrightarrow{v}\bullet \overrightarrow{\nabla }\alpha$, $\overrightarrow{v}$ is the rate of change of the displacement field $\overrightarrow{u}$ equal to $\overrightarrow{v}=\frac{\partial \overrightarrow{u}}{\partial t}$, ${\sigma}_{xy}$ is the shear stress component, A is the efficiency of conversion of stress anisotropy into structural alignment, ${\tau}_{\alpha }$ is the relaxation time of structural anisotropy, and ${D}_{\alpha }$ is the effective diffusion coefficient, i.e., non-local coupling of anisotropy. The externally imposed strain component ${\varepsilon}_{xx}$ is responsible for generation of the shear stress component ${\sigma}_{xy}$ in anisotropic inhomogeneous ventral cytoskeleton of cancer cells. Shear stress induces generation of torque governing the alignment of mesoscopic domains. This process progressively increases the degree of anisotropy α. Simultaneously, cytoskeletal remodeling causes relaxation of anisotropy represented by the second term of Eq. 2, while mechanical interactions between neighboring domains promote the spatial propagation of alignment (the third term of Eq. 2).

#### Strain components of the ventral cytoskeleton under ultrasound in the high-frequency regime

In the high-frequency regime, the storage modulus is much higher than the loss modulus, and the ventral cytoskeleton behaves as a rigid elastic grid (Fig. 2). When the ventral cytoskeleton is homogeneous (epithelial cells and fibroblasts), coupled with the structural pinning of the ventral cytoskeleton to a substrate via focal adhesions, this rigid lateral displacement constraint prevents relative horizontal deformation, forcing all in-plane strain components to vanish ${\varepsilon}_{xx}={\varepsilon}_{yy}={\varepsilon}_{xy}\approx 0$, while the degree of anisotropy is $\alpha \approx \mathrm{c}\mathrm{o}\mathrm{n}\mathrm{s}\mathrm{t}$ and leaving the out-of-plane compressional strain ${\varepsilon}_{zz}={\varepsilon}_{zz0}\mathrm{sin}\omega t$ (where ${\varepsilon}_{zz0}$ is the strain amplitude) as the sole active mode of deformation.

However, the ventral cytoskeleton of mesenchymal-like cancer cells is structurally inhomogeneous and composed of weakly connected mesodomains. Under ultrasonic excitation, these domains behave as rigid elastic grids because the microsecond oscillation period is much shorter than the characteristic times associated with cytoskeletal remodeling, stress fiber reorganization, and focal adhesion turnover (Pajic-Lijakovic et al. 2026a). Consequently, the inter-domain architecture is effectively frozen during each loading cycle. Furthermore, the widespread multi-point anchoring of focal adhesions to the substrate imposes a rigid lateral kinematic constraint, suppressing relative in-plane translations and rotations of individual domains. As a result, the in-plane strain components satisfy ${\varepsilon}_{xx}={\varepsilon}_{yy}={\varepsilon}_{xy}\approx 0$, whereas the imposed out-of-plane compressional strain, ${\varepsilon}_{zz}={\varepsilon}_{zz0}\mathrm{sin}\omega t$, induces out-of-plane tilting of the rigid mesodomains, allowing the cytoskeleton to accommodate the vertical displacement gradient (Fig. 3).

The structural rearrangement of the ventral cytoskeleton induced by external mechanical stimuli modulates the resistance to inward curvature formation around FAs. The formation of such curvature, in turn, regulates the distribution and activity of Piezo1 channels.

### Resistance effects of the ventral cytoskeleton to curvature formation around focal adhesions in the intermediate- and high-frequency regimes

The driving force for curvature formation around a FA depends on the size and activity of the FA complex. FAs consist of molecular clutches that mechanically couple the ventral cytoskeleton to the substrate matrix. The density of active molecular clutches is influenced by the stiffness of the substrate and typically ranges from 50 to 100 μm⁻^2^ (Swaminathan and Waterman 2016). Molecular clutches comprise integrins, adaptor proteins such as talin and vinculin, and the ventral cytoskeleton. The molecular clutch model was originally proposed by Chan and Odde (2008) to provide a mechanistic framework for understanding how cells sense and respond to the mechanical properties of the extracellular matrix (ECM). Consequently, the FA-induced driving force density can be expressed as: ${F}_{FA}^{out-of-plane}={\rho}_{a}\langle {f}_{c}\rangle$ (where ${\rho}_{a}$ is the surface density of active clutches and $\langle {f}_{c}\rangle$ is the average out-of-plane traction force per single molecular clutch). Oscillatory uniaxial extension in the intermediate-frequency regime stimulates the activity of FAs leading to an increase in the driving force ${F}_{FA}^{out-of-plane}$ (Tijore et al. 2021).

The viscoelastic force resisting membrane curvature formation depends on the distribution of mechanical stress around the ventral cytoskeleton, rather than on the magnitude of mechanical stress itself, and can be expressed as ${F}_{vis0}^{out-of-plane}=\overrightarrow{{\boldsymbol{n}}}\bullet {l}_{c}\nabla {\widetilde{{\boldsymbol{\sigma}}}}_{V}$ (where ${l}_{c}$ is the thickness of the ventral cytoskeleton and $\overrightarrow{{\boldsymbol{n}}}$ is the normal unit vector) (Pajic-Lijakovic et al. 2026a). Consequently, structural inhomogeneity of the ventral cytoskeleton leads to increased resistance to curvature formation around FAs. Reduced expression of tropomyosin 2.1 (TPM2.1) in mesenchymal-like cancer cells, as reported by Tijore et al. (2021), is one of the factors contributing to the inhomogeneous distribution of mechanical stress across the ventral cytoskeleton, due to impaired stabilization of actin stress fibers and weakened force transmission within the cytoskeletal network. In mesenchymal-like cancer cells, this inhomogeneity is associated with significantly higher resistance compared with epithelial cells and fibroblasts. This resistive force can exceed the driving force for curvature formation, thereby inhibiting curvature development in cancer cells, whereas curvature forms in epithelial cells and fibroblasts under uniaxial extension (Yao et al. 2022).

However, out-of-plane tilting of the ventral cytoskeleton in cancer cells under ultrasound reduces the resistive viscoelastic force to ${F}_{vis}^{out-of-plane}\approx {F}_{vis0}^{out-of-plane}\mathrm{c}\mathrm{o}\mathrm{s}\left({\theta }^{out-of-plane}\right)$ (where ${\theta }^{out-of-plane}$ is the out-of-plane tilting angle) (Pajic-Lijakovic et al. 2026a). Consequently, the viscoelastic restoring force becomes insufficient to counteract the FA-induced driving force, allowing curvature to develop in cancer cells exposed to ultrasound (Tijore et al. 2024).

While the inward curvatures around FAs in epithelial cells and fibroblasts are smooth, the curvatures around FAs in mesenchymal-like cancer cells are rough due to out-of-plane tilting of ventral cytoskeletal domains under ultrasonic load. Piezo1 distribution and activity will be considered in two regimes: the curvature-independent and curvature-dependent regimes. The properties of Piezo1 channels in both regimes depend on structural rearrangement of the ventral cytoskeleton.

## Distribution and activity of Piezo1 channels caused by altered structural changes of the ventral cytoskeleton

It is well established that Piezo1 channels preferentially accumulate in inwardly curved regions of the plasma membrane (Yang et al. 2022; Vaisey et al. 2022). The driving force which directs lateral movement of Piezo1 channels toward these curved regions is the gradient of the membrane surface tension. Piezo1 molecules migrate from flat membrane regions of lower surface tension to curved regions of higher surface tension (Pajic-Lijakovic et al. 2026b). The phenomenon represents the Marangoni effect. This effect exists in various soft matter systems, in which a surface tension gradient can be induced by changes in (i) the temperature, (ii) the distribution of constituents, or (iii) the curvature of a surface (Karbalaei et al 2016).

In epithelial cells and fibroblasts, Piezo1 clustering near FAs is observed under uniaxial extension in the intermediate-frequency regime, as well as under ultrasound loading in the high-frequency regime (Yao et al. 2022; Tijore et al. 2022; 2024). In contrast, mesenchymal-like cancer cells exhibit a more homogeneous Piezo1 distribution in the intermediate-frequency regime, whereas in the high-frequency regime Piezo1 re-localizes and clusters near FAs, similarly to the case of epithelial cells and fibroblasts (Yao et al. 2022; Tijore et al. 2024).

This behavior is consistent with the mechanical competition between FA-associated driving forces and the viscoelastic resistance of the ventral cytoskeleton (Pajic-Lijakovic et al. 2026a). In the intermediate-frequency regime, a strong viscoelastic restoring force suppresses membrane curvature formation around FAs in cancer cells, which is in accordance with experimental results from Tijore et al. (2021) and Yao et al. (2022). In the high-frequency regime, however, ultrasound-induced tilting of ventral cytoskeletal domains reduces the effective viscoelastic resistance, thereby enabling curvature formation and promoting Piezo1 clustering near FAs.

Regardless of whether curvatures form around FAs in cancer cells, Piezo1 channels become overactivated, ultimately leading to apoptosis (Yao et al. 2022; Tijore et al. 2024). In both scenarios, Piezo1 overactivation is governed by structural remodeling of the ventral cytoskeleton in response to external mechanical stimuli. This cause–effect cycle is further elaborated in the following section.

### Overactivation of Piezo1 molecules in cancer cells under uniaxial oscillatory extension

**Fig. 4:**
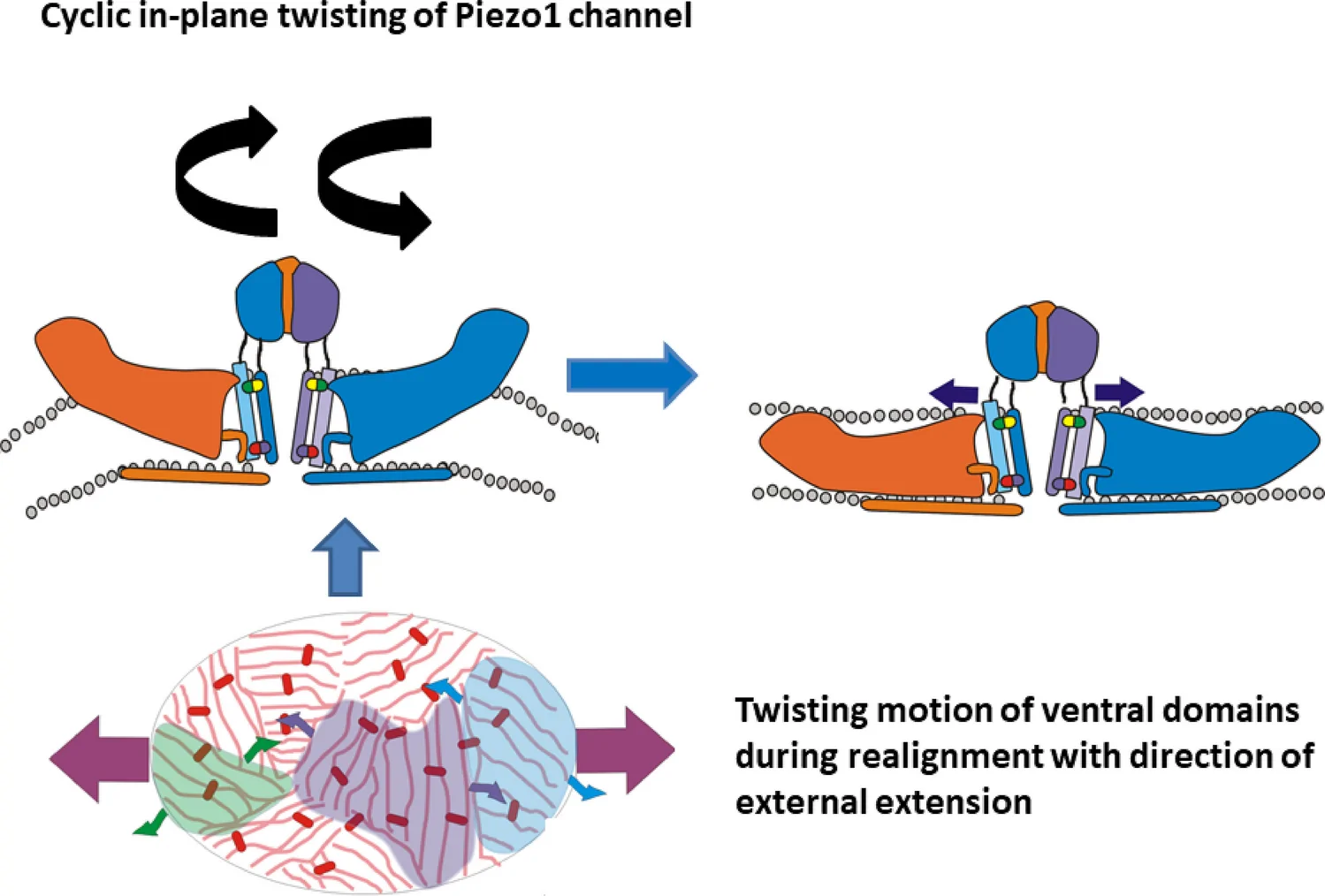
The cyclic twisting of Piezo1 channels is induced by the twisting motion of the ventral cytoskeletal domains as they realign with the direction of external uniaxial oscillatory extension

The in-plane twisting motion of ventral cytoskeletal domains in mesenchymal-like cancer cells is expected to induce cyclic rotational displacements of membrane-associated Piezo1 channels. Structural studies have shown that Piezo1 can undergo rotational conformational changes, including rotation of the extracellular cap relative to the peripheral blades (Saotome et al. 2018). More recently, Li et al. (2026), using hybrid-resolution molecular dynamics simulations supported by electrophysiological measurements, demonstrated for a structurally similar Piezo2 that flattening of the peripheral arms is coupled to rotation of the central pore relative to the arms and to clockwise twisting of the inner pore helices, leading to sequential pore dilation and hydration. Building on these findings, we propose that cyclic twisting of ventral cytoskeletal domains in mesenchymal-like cancer cells may impose a rotational mechanical input on Piezo1, potentially perturbing the conformational dynamics of its pore-forming region. Such torsional coupling may contribute to repeated Piezo1 activation. This mesoscopic alignment of ventral stress fibers is less pronounced in fibroblasts and epithelial cells. A schematic representation of the cyclic twisting of Piezo1 channel is shown in Fig. 4:

These alternating clockwise and counterclockwise rotations of the ventral cytoskeletal domain are accompanied by periodic changes in membrane surface tension, local mechanical stress, and the size and shape of the Piezo1 membrane footprint (Haselwandter and MacKinnon 2018). Under a constant external oscillation frequency, the mechanical coupling between the twisting Piezo1 channel and the surrounding lipid bilayer may give rise to a stick-slip-like response at the protein–lipid interface (Contera et al. 2005). This response depends on the lipid composition surrounding the Piezo1 channel, which is itself determined by the geometry of the Piezo1 footprint (Bavi et al. 2016; Vaisey and MacKinnon 2026).

- **Stick phase:** As the ventral cytoskeletal domain begins to rotate, the Piezo1 channel remains temporarily pinned by its interactions with the surrounding lipid bilayer. Continued twisting progressively increases the localized torsional strain and stores elastic energy at the protein–lipid interface.
- **Slip phase:** Once the accumulated torsional strain exceeds a critical threshold, the channel undergoes a rapid rotational displacement relative to the surrounding lipids, releasing the stored elastic energy. This abrupt mechanical transition can induce conformational rearrangements within Piezo1, thereby facilitating channel gating.

The abrupt release of accumulated elastic energy may trigger rapid conformational rearrangements of the Piezo1 propeller blades, thereby facilitating channel opening (Lin et al. 2019; Yang et al. 2022). The stick–slip-like response is governed by the interplay between the externally imposed oscillatory loading and local mechanical interactions at the cytoskeleton–lipid-bilayer–Piezo1 interface. We therefore propose that multiple stick–slip events could occur during a single cycle of external oscillation. Consequently, a relatively low-frequency global mechanical stimulus may generate repeated localized bursts of torsional loading and unloading at Piezo1, providing a plausible mechanism for repeated or sustained channel activation. Such prolonged Piezo1 activity is consistent with experimental observations of repetitive mechanical activation and with Piezo1 states in which inactivation is reduced or absent (Bae et al. 2013; Lewis et al. 2017).

We propose that under uniaxial oscillatory extension, ventral cytoskeletal domains undergo collective in-plane realignment accompanied by localized micro-vortical twisting. This active deformation of anisotropic domains can generate localized lipid shear waves and applies a time-dependent driving torque, $T_z$ , to the large membrane footprint of unbound, laterally migrating Piezo1 channels. The rotational response of Piezo1 is governed by the interplay of mechanical resistances acting within distinct cellular layers. The surrounding lipid bilayer acts as a threshold governed mechanical interface for the rotating Piezo1 channel. Nano-scale pinning between the channel's peripheral propeller blades and densely packed lipid domains creates an effective static friction torque, $T_{static}$ , which temporarily immobilizes the channel in its stick phase despite the continuously increasing driving torque, $T_z$ . During this phase, torsional strain and elastic potential energy progressively accumulate at the protein–lipid interface. Once the driving torque exceeds the critical threshold, the channel breaks free from the surrounding lipid environment, causing the resistive torque to decrease abruptly to a lower dynamic frictional torque, $T_{dynamic}$ , and initiating the slip phase. The rapid release of the accumulated elastic energy produces an abrupt rotational displacement of the Piezo1 channel relative to the surrounding membrane. At the same time, rotation of the channel footprint is mechanically coupled to the underlying ventral cytoskeleton. The effective rotational inertia, $I_{eff}$ , associated with elastic energy storage and characterized by the storage modulus , resists changes in angular acceleration, whereas the torsional damping coefficient, ξvis , associated with viscous energy dissipation and characterized by the loss modulus G'' V(r,ω), limits the angular velocity of the rotating channel. Inertial effects can appear as a part of low Reynolds turbulence in soft-matter systems (Datta et al. 2022; Pajic Lijakovic et al. 2024). Collective migration of epithelial monolayers also exhibits long-term inertial effects, manifested by the propagation of mechanical waves (Serra-Picamal et al. 2012). Together, these membrane- and cytoskeleton-mediated mechanical constraints generate repeated torsional loading of the Piezo1 structure, providing a plausible physical mechanism for enhanced conformational deformation of the propeller blades and facilitating channel activation under oscillatory loading. Consequently, the twisting of the Piezo1 channel caused by the in-plane twisting motion of ventral cytoskeletal domains during their realignment to uniaxial oscillatory extension can be discussed based on the balance of torques proposed here:

3 $${I}_{eff}\frac{{d}^{2}{\Delta}{\theta}_{p}}{d{t}^{2}}+{\xi}_{vis}\frac{d{\Delta}{\theta}_{p}}{dt}+{T}_{fric}\left(\frac{d{\Delta}{\theta}_{p}}{dt}\right)={T}_{z}$$

where $\Delta{\theta}_{p}$ is the relative angular displacement between Piezo1 and the ventral cytoskeleton equal to $\Delta{\theta}_{p}={\theta}_{p}-{\theta}_{v},{\theta}_{p}$ is the twisting angle of the Piezo1 channel, ${\theta}_{v}$ is the twisting angle of ventral cytoskeleton, ${I}_{eff}$ is the effective elasto-inertia of the Piezo1 channel equal to: ${I}_{eff}=\frac{1}{{\omega }^{2}}{G}_{V}{\prime}\left(r,\omega \right){R}_{F}^{2}\pi h$, ${R}_{F}$ is the radius of the Piezo1 footprint, h is the thickness of the ventral cytoskeleton, ${\xi}_{vis}$ is the torsional damping coefficient of the ventral cytoskeleton equal to ${\xi}_{vis}=\frac{1}{\omega }{G}_{V}^{{\prime}{\prime}}\left(r,\omega \right){R}_{F}^{2}\pi h$, ${R}_{F}$ is the radius of the Piezo1 footprint, h is the thickness of the ventral cytoskeleton, ${T}_{fric}\left(\frac{d{\Delta}{\theta}_{p}}{dt}\right)$ is the dry slip-stick friction torque, and ${T}_{z}$ is the applied ventral cytoskeletal torque equal to ${T}_{z}={R}_{F}^{3}{\sigma}_{Vxy}$. The dry slip-stick friction torque is (i) ${T}_{fric}\left(\frac{d{\Delta}{\theta}_{p}}{dt}\right)={T}_{static}$ for $\frac{d{\Delta}{\theta}_{p}}{dt}=0$ and (ii) ${T}_{fric}\left(\frac{d{\Delta}{\theta}_{p}}{dt}\right)={T}_{dynmic}sgn(\frac{d{\Delta}{\theta}_{p}}{dt})$ for $\frac{d{\Delta}{\theta}_{p}}{dt}\ne 0$ (where static torque and dynamic torque satisfy the condition that ${T}_{static}>\left|{T}_{dynmic}\right|$.

In further consideration, it is necessary to identify the cause of Piezo1 overactivation in cancer cells under ultrasound loading and relate it to the structural rearrangement of the ventral cytoskeleton in the high-frequency regime.

### Overactivation of Piezo1 molecules in cancer cells under ultrasound load

Piezo1 channels cluster in the curved regions around FAs in epithelial cells and fibroblasts under oscillatory extension in the intermediate-frequency range and under ultrasound loading in the high-frequency range (Tijore et al. 2021; Yao et al. 2022). In contrast, in mesenchymal-like cancer cells, Piezo1 clusters around FAs are formed only under ultrasound loading (Tijore et al. 2024). Although Piezo1 channels cluster around FAs in all cell types in the high-frequency regime, they become overactivated only in cancer cells. These observations indicate that Piezo1 clustering within curved regions around FAs under ultrasound is a necessary, but not sufficient, condition for channel overactivation, implying that an additional mechanism is required to promote excessive Piezo1 activation in cancer cells (Pajic-Lijakovic et al. 2026a). This mechanism is related to the roughness of curved regions.

While curved regions are smooth in epithelial cells and fibroblasts, they are rough in mesenchymal-like cancer cells because of the out-of-plane tilting of the ventral cytoskeletal domains (Pajic-Lijakovic et al. 2026a). Although membrane curvature does not affect the footprint of an isolated Piezo1 channel, it influences the coupling between Piezo1 molecules within clusters (Bavi et al. 2016; 2019), thereby modulating channel activity (Pajic-Lijakovic et al. 2026a). Piezo1 preferentially adopts an outward membrane footprint, in which the surrounding lipids stabilize mechanically sensitive conformations while allowing the protein–lipid complex to flatten readily under mechanical loading (Vaisey and MacKinnon 2026). Consequently, the outward footprint represents the mechanically preferred state for efficient Piezo1 gating (Bavi et al. 2016).

Cholesterol further stabilizes this preferred outward footprint through both direct binding to Piezo1 and indirect modulation of membrane mechanics (Ridone et al. 2019; Zakany et al. 2020). Direct cholesterol binding stabilizes the anchor region and promotes long-lived lipid–protein interactions (Buyan et al. 2020), whereas indirect membrane stiffening increases the energy barrier for conformational transitions (Zakany et al. 2020). Because cholesterol is heterogeneously distributed within the plasma membrane, it creates microdomains with distinct mechanical properties that locally regulate Piezo1 activity. In contrast, transition to an inward footprint weakens cholesterol binding, softens the surrounding membrane, destabilizes Piezo1 clusters, and suppresses channel gating (Ridone et al. 2019; Short 2020).

**Fig. 5:**
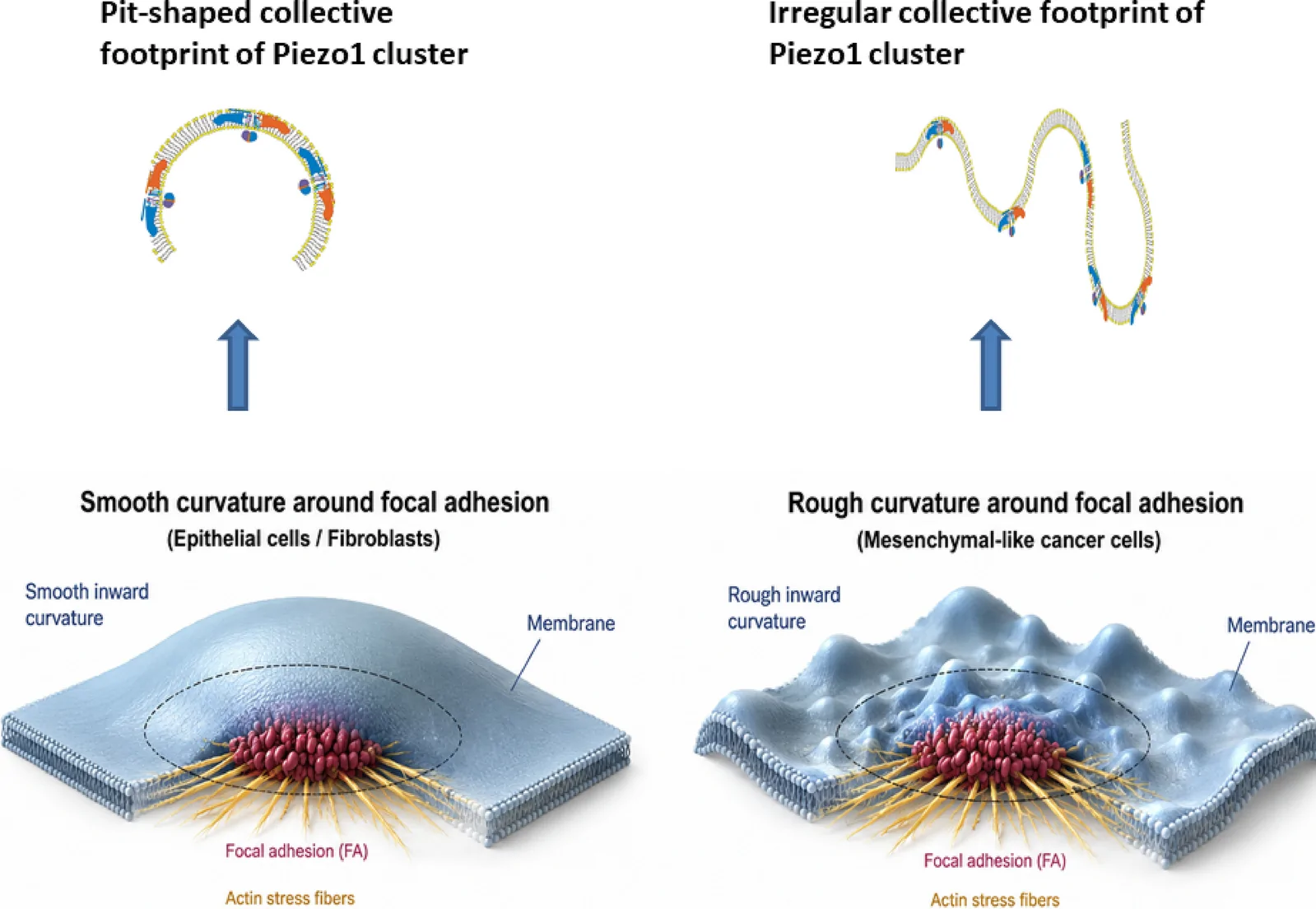
The pit-shaped collective footprint of a Piezo1 cluster in the smooth curved region around a FA in epithelial cells and fibroblasts (left) is contrasted with the irregular collective footprint of a Piezo1 cluster in the rough curved region around a FA in cancer cells (right). While a pit-shaped collective footprint reduces Piezo1 activity, an irregular collective footprint is the main cause of overactivation of Piezo1 molecules

While smooth inward curvature promotes a transition of Piezo1 footprints from the preferred outward configuration to an inward state, leading to the formation of pit-shaped invaginations enriched in Piezo1 clusters (Verkest et al., 2026), this process is altered in cancer cells because the curvature around FAs is rough and spatially heterogeneous due to the out-of-plane tilting of the underlying ventral cytoskeletal domains. The lipid bilayer follows these local cytoskeletal deformations, resulting in spatially varying curvature orientations within a Piezo1 cluster. Consequently, neighboring Piezo1 molecules can experience locally different curvature orientations, with some adopting inward-oriented footprints and others retaining the preferred outward-oriented footprints, as shown schematically in Fig. 5:

Geometrical noise associated with the rough curvature around FAs can preserve the outward footprint of Piezo1 channels within clusters (Pajic-Lijakovic et al. 2026a). Thus, whereas smooth curvature drives a cooperative inward remodeling of a Piezo1 cluster, rough curvature stabilizes the outward footprint and prevents full pit-like reorganization of the cluster enhancing the activity of Piezo1 channels (Pajic-Lijakovic et al. 2026a).

The collective footprint of a Piezo1 cluster in the curved region around a FA results from a minimization of the surface free energy: $\frac{\delta {E}_{S}}{\delta h}=0$ (where $h\left(r,t\right)$ is the membrane height around the FA and ${E}_{S}\left(r,t\right)$ is the surface free energy). The surface free energy can be expressed (Helfrich 1973; Pajic-Lijakovic et al. 2026d) as:

4 $${E}_{S}\left(r,t\right)=\int \left[\frac{\kappa }{2}{\left({\overrightarrow{\nabla }}^{2}h-{C}_{FA}\right)}^{2}+\frac{{\gamma}_{m}}{2}{\left(\overrightarrow{\nabla }h\right)}^{2}\right]{d}^{2}r+{\int }\frac{\kappa }{2}{{C}_{p}}^{2}{d}^{2}r+{\int }\kappa \left({\overrightarrow{\nabla }}^{2}h-{C}_{FA}\right){C}_{p}{d}^{2}{r}$$

where ${C}_{FA}$ is the mesoscopic curvature around the FA, ${C}_{p}$ is the collective footprint of the Piezo1 cluster, κ is the bending modulus of the lipid bilayer, and ${\gamma}_{m}$ is the membrane surface tension. Three contributions to the surface free energy ${E}_{S}$ can be identified. The first term represents the baseline membrane energy in the vicinity of the FA in the absence of Piezo1-induced perturbations. The second term is the intrinsic self-energy of the Piezo1 cluster, which sets the energetic cost of forming and maintaining the outward footprint and defines the barrier for the outward-to-inward transition. The third term is the curvature–footprint coupling term, which governs how effectively Piezo1 footprints align with FA-induced curvature.

The balance between these contributions depends critically on the nature of the curvature field. In smooth curvature landscapes, ${C}_{FA}$ varies slowly in space, minimizing the baseline membrane energy and establishing a coherent mechanical template. In this regime, the coupling term remains spatially correlated with ${\overrightarrow{\nabla }}^{2}h-{C}_{FA}$, producing a systematic energy gain that can overcome the self-energy barrier and drive a transition toward inward, pit-like Piezo1 organization. In contrast, rough curvature fields introduce strong spatial fluctuations in ${C}_{FA}$, generating a mechanically frustrated membrane environment. The resulting rapid sign changes in ${\overrightarrow{\nabla }}^{2}h-{C}_{FA}$ lead to stochastic cancelation of the coupling term when integrated over ${C}_{p}$, rendering it ineffective. Consequently, the self-energy dominates, locking Piezo1 clusters into their high-energy outward footprint and preventing collective inversion even at high cluster density.

## Conclusion

This review establishes a unified mechanobiological framework describing how oscillatory mechanical stimuli induce Piezo1 overactivation in mesenchymal-like cancer cells through frequency-dependent remodeling of the ventral cytoskeleton. The central concept emerging from the analysis is that Piezo1 responds directly to mechanical perturbations of the membrane; however, the activity of the channel is critically regulated by the structural and mechanical state of the basal membrane–ventral cytoskeleton system. Through frequency-dependent remodeling of the ventral cytoskeleton, external mechanical inputs are spatially redistributed and converted into localized changes in membrane curvature, protein–lipid coupling, and Piezo1 conformational dynamics.

The enhanced mechanical vulnerability of mesenchymal-like cancer cells originates from the unique organization of their ventral cytoskeleton, characterized by structural heterogeneity and anisotropy. In contrast to the homogeneous cytoskeletal networks of epithelial cells and fibroblasts, cancer cells contain weakly connected mesoscopic domains with distinct orientations, mechanical properties, and relaxation dynamics. This domain-based architecture promotes spatially heterogeneous stress redistribution and enables mechanical responses that depend strongly on the frequency and nature of the external loading. The hierarchical viscoelasticity of the ventral cytoskeleton, governed by both the intrinsic properties of stress fibers and their interconnections, determines the balance between elastic energy storage and viscous dissipation, thereby controlling how mechanical forces are transmitted to the membrane and Piezo1 channels.

Two complementary mechanisms of Piezo1 overactivation emerge from the analysis of different oscillatory loading regimes. Under intermediate-frequency oscillatory extension, anisotropic cytoskeletal domains generate shear stresses and torque-like imbalances that induce in-plane twisting and progressive realignment of cytoskeletal domains. This rotational remodeling provides a mechanism by which a relatively slow global deformation can be converted into localized torsional loading of membrane-associated Piezo1 channels. The proposed stick–slip-like interaction between Piezo1 and the surrounding lipid environment provides a plausible explanation for repeated cycles of torsional energy accumulation and release, which may promote persistent conformational deformation of Piezo1 and sustained channel activation.

Under high-frequency ultrasound stimulation, the short oscillation period prevents substantial cytoskeletal reorganization within individual loading cycles, causing the ventral cytoskeleton to behave as an elastic network of mechanically constrained domains. Ultrasound-induced out-of-plane tilting of these domains reduces effective resistance to focal adhesion-associated membrane deformation and enables the formation of rough membrane curvatures around focal adhesions. Importantly, Piezo1 clustering alone is insufficient to explain channel overactivation. The mechanical characteristics of the curvature landscape are critical: smooth curvatures promote cooperative footprint remodeling of Piezo1 clusters, whereas rough curvatures generated by cytoskeletal heterogeneity can stabilize mechanically sensitive outward footprints and enhance channel activity.

Collectively, these findings indicate that Piezo1 overactivation emerges from the coupled mechanics of the ventral cytoskeleton, membrane geometry, focal adhesions, and protein–lipid interactions. The frequency and amplitude of external mechanical stimuli determine the extent and mode of structural remodeling within these interconnected cellular components, thereby modifying their mechanical coupling and creating feedback regulation of Piezo1 activity. Thus, Piezo1 activation is governed not only by the characteristics of the applied mechanical stimulus but also by the dynamically remodeled mechanical state of the basal membrane–cytoskeleton system. The proposed framework provides a physical explanation for why mechanically identical stimuli can generate distinct biological outcomes in different cell types and identifies cytoskeletal architecture as a key determinant of cancer cell mechanical vulnerability. This perspective offers a basis for designing mechanically selective therapeutic approaches that exploit the unique viscoelastic and structural properties of cancer cells while minimizing effects on healthy tissues. Future experimental studies combining quantitative measurements of cytoskeletal domain dynamics, membrane curvature evolution, Piezo1 conformational transitions, and controlled mechanical stimulation will be essential to validate and refine this mechanistic model.

## Data Availability

No datasets were generated or analysed during the current study.
